# Bis{μ-1-[(2-ethyl-1*H*-imidazol-1-yl)meth­yl]-1*H*-benzotriazole}bis­(iodido­cadmium)

**DOI:** 10.1107/S1600536811021179

**Published:** 2011-06-11

**Authors:** Xia Wang, Jun-long Niu

**Affiliations:** a Pharmacy College, Henan University of Traditional Chinese Medicine, Zhengzhou 450008, People’s Republic of China; b Department of Chemistry, Henan Key Laboratory of Chemical Biology and Organic Chemistry, Zhengzhou University, Zhengzhou 450052, People’s Republic of China

## Abstract

The dinuclear title complex, [Cd_2_I_4_(C_12_H_13_N_5_)_2_], lies on a crystallographic center of inversion. The Cd^II^ atom is four-coordinated by two N atoms from two 1-[(2-ethyl-1*H*-imidazol-1-yl)meth­yl]-1*H*-benzotriazole (bmei) ligands and two terminal I atoms in a distorted tetra­hedral coordination environment. The Cd^II^ atoms are connected to each other by two bridging bmei ligands. The benzotriazole rings in adjacent mol­ecules are almost parallel, with an average inter­planar distance of 3.3400 (2) Å and a centroid–centroid distance of 4.852 (2) Å.

## Related literature

For related structures, see: Meng *et al.* (2009 ▶); Huang *et al.* (2006 ▶); Zhai *et al.* (2006 ▶); Wang *et al.* (2010 ▶).
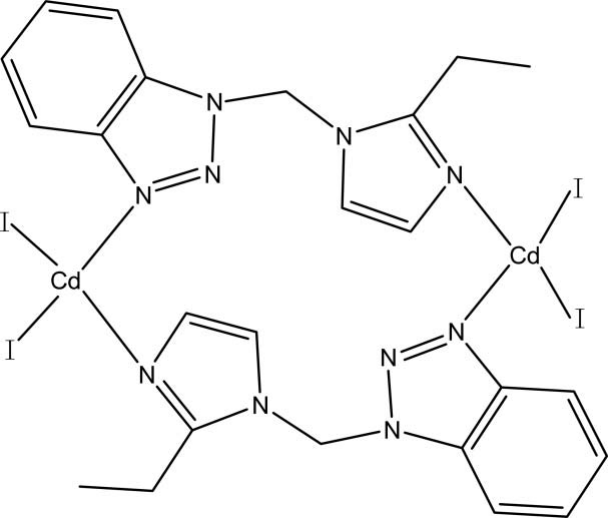


## Experimental

### 

#### Crystal data

[Cd_2_I_4_(C_12_H_13_N_5_)_2_]
*M* *_r_* = 1186.95
Triclinic, 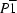

*a* = 7.8323 (4) Å
*b* = 10.0657 (6) Å
*c* = 11.2335 (7) Å
α = 78.849 (5)°
β = 86.020 (5)°
γ = 77.538 (5)°
*V* = 848.08 (9) Å^3^
*Z* = 1
Mo *K*α radiation
μ = 4.93 mm^−1^
*T* = 290 K
0.25 × 0.21 × 0.15 mm

#### Data collection

Agilent Xcalibur Eos Gemini diffractometer
Absorption correction: Gaussian [numerical absorption correction based on Gaussian integration over a multifaceted crystal model (*CrysAlis PRO*; Agilent, 2010 ▶)] *T*_min_ = 0.287, *T*_max_ = 0.487
13998 measured reflections
3462 independent reflections
2961 reflections with *I* > 2σ(*I*)
*R*_int_ = 0.031

#### Refinement

*R*[*F* ^2^ > 2σ(*F* ^2^)] = 0.027
*wR*(*F* ^2^) = 0.061
*S* = 1.08
3462 reflections
182 parameters
H-atom parameters constrained
Δρ_max_ = 0.82 e Å^−3^
Δρ_min_ = −1.05 e Å^−3^

### 

Data collection: *CrysAlis PRO* (Agilent, 2010 ▶); cell refinement: *CrysAlis PRO*; data reduction: *CrysAlis PRO*; program(s) used to solve structure: *SHELXS97* (Sheldrick, 2008 ▶); program(s) used to refine structure: *SHELXL97* (Sheldrick, 2008 ▶); molecular graphics: *OLEX2* (Dolomanov *et al.*, 2009 ▶); software used to prepare material for publication: *OLEX2* and *publCIF* (Westrip, 2010 ▶).

## Supplementary Material

Crystal structure: contains datablock(s) global, I. DOI: 10.1107/S1600536811021179/zq2107sup1.cif

Structure factors: contains datablock(s) I. DOI: 10.1107/S1600536811021179/zq2107Isup2.hkl

Additional supplementary materials:  crystallographic information; 3D view; checkCIF report

## Figures and Tables

**Fig. 1.**
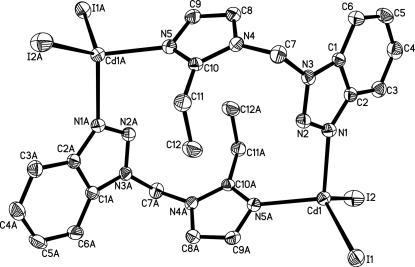
View of the title complex, showing the labeling of the 30% probability ellipsolids (all H atoms have been omitted for clarity). Symmetry code: (A) -x + 1, -y + 2, -z.

**Fig. 2.**
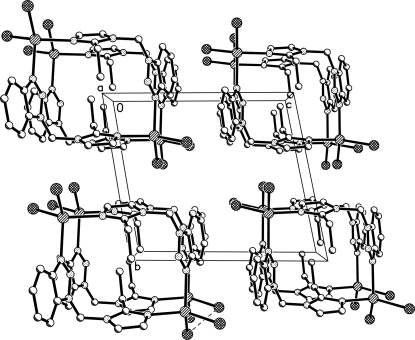
A view of the crystal packing along the a axis. All H atoms are omitted for clarity.

## supplementary crystallographic information

### Comment

Imidazole and benzotriazole derivatives have been widely used in the construction of complexes since they can act as polydentate ligands and function as bridging ligands (Meng *et al.*, 2009; Huang *et al.*, 2006). The Cd^II^ atom is a good model atom to construct complexes owing to its property to form bonds with different donors simultaneously, and to its various modes (Zhai *et al.*, 2006; Wang *et al.*, 2010). In this work, through the reaction of 1-[(2-ethyl-1*H*-imidazol-1-yl)methyl]-1*H*-benzotriazole (bmei) with cadmium iodide at room temperature, we obtained the title complex [Cd_2_(C_12_H_13_N_5_)_2_I_2_], which is reported here.

The dinuclear title complex, [Cd_2_(C_12_H_13_N_5_)_2_I_2_], lies on a crystallographic center of inversion. The Cd^II^ atom is four-coordinated by two N atoms from two 1-[(2-ethyl-1*H*-imidazol-1-yl)methyl]-1*H*-benzotriazole (bmei) ligands and two terminal I atoms in a distorted tetrahedral coordination environment. The Cd^II^ atoms are connected by two bridging bmei ligands (Fig. 1). The distance between two Cd atoms bridged by two bmei ligands is 8.4983 (7) Å. In addition, the benzotriazole rings in adjacent molecules are almost parallel with an average interplanar distance of 3.3400 (2) Å and a centroid-centroid distance of 4.852 (2) Å.

### Experimental

The ligand 1-[(2-ethyl-1*H*-imidazol-1-yl)methyl]-1*H*-benzotriazole (0.04 mmol, 0.0096 g) in methanol (6 ml) was added dropwise to a methanol solution (6 ml) of CdI2 (0.04 mmol, 0.0146 g) in methanol. The resulting solution was allowed to stand at room temperature. After two weeks good quality colourless crystals were obtained from the dried in air.

### Refinement

H atoms were generated geometrically and refined as riding atoms with C—H = 0.93 Å and *U*_iso_(H) = 1.2*U*_eq_(C) for aromatic H atoms, with C—H = 0.97 Å and *U*_iso_(H) = 1.2*U*_eq_(C) for methylene H atoms, and with C—H = 0.96 Å and *U*_iso_(H) = 1.5*U*_eq_(C) for methyl H atoms.

### Crystal data

**Table:**

[Cd_2_I_4_(C_12_H_13_N_5_)_2_]	*Z* = 1
*M**_r_* = 1186.95	*F*(000) = 548
Triclinic, *P*1	*D*_x_ = 2.324 Mg m^−^^3^
*a* = 7.8323 (4) Å	Mo *K*α radiation, λ = 0.7107 Å
*b* = 10.0657 (6) Å	Cell parameters from 6849 reflections
*c* = 11.2335 (7) Å	θ = 3.0–26.3°
α = 78.849 (5)°	µ = 4.93 mm^−^^1^
β = 86.020 (5)°	*T* = 290 K
γ = 77.538 (5)°	Prismatic, colourless
*V* = 848.08 (9) Å^3^	0.25 × 0.21 × 0.15 mm

### Data collection

**Table:**

Agilent Xcalibur Eos Gemini diffractometer	3462 independent reflections
Radiation source: Enhance (Mo) X-ray Source	2961 reflections with *I* > 2σ(*I*)
graphite	*R*_int_ = 0.031
Detector resolution: 16.2312 pixels mm^-1^	θ_max_ = 26.3°, θ_min_ = 3.0°
ω scans	*h* = −9→9
Absorption correction: gaussian [numerical absorption correction based on Gaussian integration over a multifaceted crystal model (*CrysAlis PRO*; Agilent, 2010)]	*k* = −12→12
*T*_min_ = 0.287, *T*_max_ = 0.487	*l* = −14→14
13998 measured reflections	

### Refinement

**Table:**

Refinement on *F*^2^	Primary atom site location: structure-invariant direct methods
Least-squares matrix: full	Secondary atom site location: difference Fourier map
*R*[*F*^2^ > 2σ(*F*^2^)] = 0.027	Hydrogen site location: inferred from neighbouring sites
*wR*(*F*^2^) = 0.061	H-atom parameters constrained
*S* = 1.08	*w* = 1/[σ^2^(*F*_o_^2^) + (0.026*P*)^2^ + 0.3471*P*] where *P* = (*F*_o_^2^ + 2*F*_c_^2^)/3
3462 reflections	(Δ/σ)_max_ = 0.002
182 parameters	Δρ_max_ = 0.82 e Å^−^^3^
0 restraints	Δρ_min_ = −1.05 e Å^−^^3^

### Special details

**Table:**

Geometry. All e.s.d.'s (except the e.s.d. in the dihedral angle between two l.s. planes) are estimated using the full covariance matrix. The cell e.s.d.'s are taken into account individually in the estimation of e.s.d.'s in distances, angles and torsion angles; correlations between e.s.d.'s in cell parameters are only used when they are defined by crystal symmetry. An approximate (isotropic) treatment of cell e.s.d.'s is used for estimating e.s.d.'s involving l.s. planes.
Refinement. Refinement of *F*^2^ against ALL reflections. The weighted *R*-factor *wR* and goodness of fit *S* are based on *F*^2^, conventional *R*-factors *R* are based on *F*, with *F* set to zero for negative *F*^2^. The threshold expression of *F*^2^ > σ(*F*^2^) is used only for calculating *R*-factors(gt) *etc*. and is not relevant to the choice of reflections for refinement. *R*-factors based on *F*^2^ are statistically about twice as large as those based on *F*, and *R*- factors based on ALL data will be even larger.

### Fractional atomic coordinates and isotropic or equivalent isotropic displacement parameters (Å^2^)

**Table:**

	*x*	*y*	*z*	*U*_iso_*/*U*_eq_	
I1	0.49488 (4)	1.31204 (3)	0.40337 (2)	0.04897 (9)	
I2	−0.04748 (4)	1.42680 (3)	0.22432 (3)	0.06153 (11)	
Cd1	0.27758 (4)	1.26989 (3)	0.24333 (2)	0.03657 (9)	
N1	0.2561 (4)	1.0409 (3)	0.2834 (3)	0.0385 (7)	
N2	0.4017 (4)	0.9559 (3)	0.2627 (3)	0.0389 (7)	
N3	0.3713 (4)	0.8276 (3)	0.2862 (3)	0.0350 (7)	
N4	0.5058 (4)	0.7062 (3)	0.1294 (3)	0.0354 (7)	
N5	0.5744 (4)	0.7249 (3)	−0.0641 (3)	0.0370 (7)	
C1	0.2006 (5)	0.8280 (4)	0.3244 (3)	0.0343 (8)	
C2	0.1274 (5)	0.9679 (4)	0.3215 (3)	0.0356 (8)	
C3	−0.0491 (5)	1.0113 (4)	0.3544 (4)	0.0451 (10)	
H3	−0.1003	1.1043	0.3512	0.054*	
C4	−0.1422 (6)	0.9082 (5)	0.3916 (4)	0.0514 (11)	
H4	−0.2598	0.9322	0.4139	0.062*	
C5	−0.0643 (6)	0.7676 (5)	0.3966 (4)	0.0525 (11)	
H5	−0.1322	0.7018	0.4236	0.063*	
C6	0.1076 (6)	0.7234 (4)	0.3635 (4)	0.0469 (10)	
H6	0.1583	0.6303	0.3670	0.056*	
C7	0.5056 (5)	0.7136 (4)	0.2573 (3)	0.0421 (9)	
H7A	0.4851	0.6274	0.3059	0.051*	
H7B	0.6192	0.7258	0.2772	0.051*	
C8	0.3756 (5)	0.6688 (4)	0.0752 (4)	0.0432 (9)	
H8	0.2762	0.6414	0.1132	0.052*	
C9	0.4206 (5)	0.6798 (4)	−0.0435 (4)	0.0440 (9)	
H9	0.3570	0.6598	−0.1023	0.053*	
C10	0.6246 (5)	0.7411 (3)	0.0414 (3)	0.0330 (8)	
C11	0.7851 (5)	0.7927 (4)	0.0590 (4)	0.0442 (9)	
H11B	0.8741	0.7642	−0.0007	0.053*	
H11A	0.8297	0.7494	0.1388	0.053*	
C12	0.7549 (6)	0.9494 (4)	0.0479 (4)	0.0564 (11)	
H12B	0.7143	0.9933	−0.0316	0.085*	
H12C	0.8626	0.9748	0.0607	0.085*	
H12A	0.6687	0.9786	0.1078	0.085*	

### Atomic displacement parameters (Å^2^)

**Table:**

	*U*^11^	*U*^22^	*U*^33^	*U*^12^	*U*^13^	*U*^23^
I1	0.04834 (17)	0.06438 (19)	0.03873 (15)	−0.02051 (14)	−0.00434 (12)	−0.00950 (13)
I2	0.04034 (16)	0.04634 (17)	0.0956 (3)	−0.00025 (13)	−0.00964 (16)	−0.01437 (16)
Cd1	0.03734 (16)	0.03522 (15)	0.03821 (16)	−0.00826 (12)	0.00067 (12)	−0.00907 (12)
N1	0.0398 (18)	0.0340 (17)	0.0428 (18)	−0.0096 (14)	0.0045 (15)	−0.0097 (14)
N2	0.0433 (18)	0.0328 (17)	0.0398 (17)	−0.0086 (14)	0.0052 (15)	−0.0065 (14)
N3	0.0410 (17)	0.0326 (16)	0.0298 (15)	−0.0068 (14)	0.0026 (13)	−0.0037 (12)
N4	0.0390 (17)	0.0341 (16)	0.0343 (16)	−0.0070 (14)	0.0003 (14)	−0.0103 (13)
N5	0.0422 (18)	0.0361 (17)	0.0344 (17)	−0.0120 (14)	0.0013 (14)	−0.0074 (13)
C1	0.039 (2)	0.038 (2)	0.0278 (18)	−0.0134 (17)	−0.0008 (16)	−0.0064 (15)
C2	0.039 (2)	0.040 (2)	0.0297 (18)	−0.0114 (17)	0.0007 (16)	−0.0074 (16)
C3	0.040 (2)	0.050 (2)	0.047 (2)	−0.0078 (19)	0.0020 (19)	−0.0138 (19)
C4	0.038 (2)	0.071 (3)	0.049 (2)	−0.019 (2)	0.0026 (19)	−0.015 (2)
C5	0.053 (3)	0.061 (3)	0.053 (3)	−0.036 (2)	0.004 (2)	−0.007 (2)
C6	0.058 (3)	0.042 (2)	0.045 (2)	−0.020 (2)	0.003 (2)	−0.0083 (18)
C7	0.046 (2)	0.038 (2)	0.038 (2)	0.0005 (18)	−0.0032 (18)	−0.0041 (17)
C8	0.042 (2)	0.042 (2)	0.050 (2)	−0.0147 (18)	0.0083 (19)	−0.0142 (19)
C9	0.044 (2)	0.047 (2)	0.049 (2)	−0.0176 (19)	0.0011 (19)	−0.0195 (19)
C10	0.0353 (19)	0.0265 (17)	0.037 (2)	−0.0068 (15)	0.0012 (16)	−0.0058 (15)
C11	0.039 (2)	0.051 (2)	0.046 (2)	−0.0116 (19)	−0.0028 (18)	−0.0127 (19)
C12	0.055 (3)	0.059 (3)	0.062 (3)	−0.028 (2)	−0.001 (2)	−0.011 (2)

### Geometric parameters (Å, °)

**Table:**

I1—Cd1	2.7094 (4)	C3—C4	1.379 (6)
I2—Cd1	2.6892 (4)	C4—H4	0.9300
Cd1—N1	2.302 (3)	C4—C5	1.406 (6)
Cd1—N5^i^	2.250 (3)	C5—H5	0.9300
N1—N2	1.306 (4)	C5—C6	1.373 (6)
N1—C2	1.371 (5)	C6—H6	0.9300
N2—N3	1.337 (4)	C7—H7A	0.9700
N3—C1	1.375 (5)	C7—H7B	0.9700
N3—C7	1.451 (5)	C8—H8	0.9300
N4—C7	1.452 (5)	C8—C9	1.345 (5)
N4—C8	1.376 (5)	C9—H9	0.9300
N4—C10	1.359 (5)	C10—C11	1.499 (5)
N5—Cd1^i^	2.250 (3)	C11—H11B	0.9700
N5—C9	1.367 (5)	C11—H11A	0.9700
N5—C10	1.323 (4)	C11—C12	1.525 (6)
C1—C2	1.396 (5)	C12—H12B	0.9600
C1—C6	1.393 (5)	C12—H12C	0.9600
C2—C3	1.403 (5)	C12—H12A	0.9600
C3—H3	0.9300		
			
I2—Cd1—I1	118.809 (14)	C4—C3—C2	116.1 (4)
N1—Cd1—I1	109.18 (8)	C4—C3—H3	121.9
N1—Cd1—I2	108.30 (8)	C4—C5—H5	118.6
N1—N2—N3	107.9 (3)	C5—C4—H4	119.1
N1—C2—C1	107.6 (3)	C5—C6—C1	115.2 (4)
N1—C2—C3	131.4 (4)	C5—C6—H6	122.4
N2—N1—Cd1	113.7 (2)	C6—C1—C2	123.0 (4)
N2—N1—C2	109.7 (3)	C6—C5—C4	122.8 (4)
N2—N3—C1	111.1 (3)	C6—C5—H5	118.6
N2—N3—C7	119.6 (3)	H7A—C7—H7B	108.0
N3—C1—C2	103.7 (3)	C8—N4—C7	124.5 (3)
N3—C1—C6	133.2 (4)	C8—C9—N5	109.3 (4)
N3—C7—N4	110.9 (3)	C8—C9—H9	125.4
N3—C7—H7A	109.5	C9—N5—Cd1^i^	122.4 (3)
N3—C7—H7B	109.5	C9—C8—N4	106.4 (4)
N4—C7—H7A	109.5	C9—C8—H8	126.8
N4—C7—H7B	109.5	C10—N4—C7	127.6 (3)
N4—C8—H8	126.8	C10—N4—C8	107.8 (3)
N4—C10—C11	126.0 (3)	C10—N5—Cd1^i^	129.4 (3)
N5^i^—Cd1—I1	106.59 (8)	C10—N5—C9	107.5 (3)
N5^i^—Cd1—I2	112.86 (8)	C10—C11—H11B	108.7
N5^i^—Cd1—N1	99.31 (11)	C10—C11—H11A	108.7
N5—C9—H9	125.4	C10—C11—C12	114.1 (3)
N5—C10—N4	109.1 (3)	C11—C12—H12B	109.5
N5—C10—C11	124.9 (3)	C11—C12—H12C	109.5
C1—N3—C7	128.9 (3)	C11—C12—H12A	109.5
C1—C2—C3	121.0 (4)	H11B—C11—H11A	107.6
C1—C6—H6	122.4	C12—C11—H11B	108.7
C2—N1—Cd1	136.6 (2)	C12—C11—H11A	108.7
C2—C3—H3	121.9	H12B—C12—H12C	109.5
C3—C4—H4	119.1	H12B—C12—H12A	109.5
C3—C4—C5	121.9 (4)	H12C—C12—H12A	109.5
			
I1—Cd1—N1—N2	−66.6 (2)	N5^i^—Cd1—N1—C2	−133.4 (4)
I1—Cd1—N1—C2	115.3 (3)	N5—C10—C11—C12	90.2 (5)
I2—Cd1—N1—N2	162.7 (2)	C1—N3—C7—N4	90.9 (4)
I2—Cd1—N1—C2	−15.4 (4)	C1—C2—C3—C4	1.4 (6)
Cd1—N1—N2—N3	−178.4 (2)	C2—N1—N2—N3	0.2 (4)
Cd1—N1—C2—C1	178.3 (3)	C2—C1—C6—C5	1.6 (6)
Cd1—N1—C2—C3	−1.7 (6)	C2—C3—C4—C5	0.2 (6)
Cd1^i^—N5—C9—C8	171.0 (2)	C3—C4—C5—C6	−1.0 (7)
Cd1^i^—N5—C10—N4	−169.5 (2)	C4—C5—C6—C1	0.1 (6)
Cd1^i^—N5—C10—C11	11.7 (5)	C6—C1—C2—N1	177.6 (3)
N1—N2—N3—C1	−0.5 (4)	C6—C1—C2—C3	−2.4 (6)
N1—N2—N3—C7	172.9 (3)	C7—N3—C1—C2	−172.0 (3)
N1—C2—C3—C4	−178.6 (4)	C7—N3—C1—C6	10.3 (7)
N2—N1—C2—C1	0.1 (4)	C7—N4—C8—C9	177.7 (3)
N2—N1—C2—C3	−179.9 (4)	C7—N4—C10—N5	−177.4 (3)
N2—N3—C1—C2	0.5 (4)	C7—N4—C10—C11	1.3 (6)
N2—N3—C1—C6	−177.1 (4)	C8—N4—C7—N3	−69.3 (5)
N2—N3—C7—N4	−81.1 (4)	C8—N4—C10—N5	−0.9 (4)
N3—C1—C2—N1	−0.4 (4)	C8—N4—C10—C11	177.9 (3)
N3—C1—C2—C3	179.6 (3)	C9—N5—C10—N4	0.4 (4)
N3—C1—C6—C5	178.9 (4)	C9—N5—C10—C11	−178.4 (3)
N4—C8—C9—N5	−0.8 (4)	C10—N4—C7—N3	106.8 (4)
N4—C10—C11—C12	−88.4 (5)	C10—N4—C8—C9	1.0 (4)
N5^i^—Cd1—N1—N2	44.7 (3)	C10—N5—C9—C8	0.3 (4)

Symmetry codes: (i) −*x*+1, −*y*+2, −*z*.
